# Mulberry Fruit Cultivar ‘Chiang Mai’ Prevents Beta-Amyloid Toxicity in PC12 Neuronal Cells and in a *Drosophila* Model of Alzheimer’s Disease

**DOI:** 10.3390/molecules25081837

**Published:** 2020-04-15

**Authors:** Uthaiwan Suttisansanee, Somsri Charoenkiatkul, Butsara Jongruaysup, Somying Tabtimsri, Dalad Siriwan, Piya Temviriyanukul

**Affiliations:** 1 Institute of Nutrition, Mahidol University, Salaya, Phuttamonthon, Nakhon Pathom 73170, Thailand; uthaiwan.sut@mahidol.ac.th (U.S.); somsri.chr@mahidol.ac.th (S.C.); 2 Office of Sericulture Conservation and Standard Conformity Assessment, The Queen Sirikit Department of Sericulture, Ministry of Agriculture and Cooperatives, Bangkok 10900, Thailand; butsara_2000@hotmail.com; 3 The Queen Sirikit Department of Sericulture Center (Kanchanaburi), Nong Ya, Mueang Kanchanaburi District, Kanchanaburi 71000, Thailand; yodyingtts@gmail.com; 4 Institute of Food Research and Product Development, Kasetsart University, Chatuchak, Bangkok 10900, Thailand

**Keywords:** *Morus* species, Alzheimer’s disease, anthocyanins, anthocyanidins, amyloid peptides, beta-secretase 1, *Drosophila melanogaster*

## Abstract

Alzheimer’s disease (AD) is the most common form of dementia, characterized by chronic neuron loss and cognitive problems. Aggregated amyloid beta (Aβ) peptides, a product of cleaved amyloid precursor protein (APP) by beta-secretase 1 (BACE-1), have been indicated for the progressive pathogenesis of AD. Currently, screening for anti-AD compounds in foodstuffs is increasing, with promising results. Hence, the purpose of this study was to investigate the extraction conditions, phytochemical contents, and anti-AD properties, targeting Aβ peptides of *Morus* cf. *nigra* ‘Chiang Mai’ (MNCM) both in vitro and in vivo. Data showed that the aqueous extract of MNCM contained high amounts of cyanidin, keracyanin, and kuromanin as anthocyanidin and anthocyanins. The extract also strongly inhibited cholinesterases and BACE-1 in vitro. Moreover, MNCM extract prevented Aβ-induced neurotoxicity and promoted neurite outgrowth in neuronal cells. Interestingly, MNCM extract reduced Aβ_1–42_ peptides and improved locomotory coordination of *Drosophila* co-expressing human APP and BACE-1, specifically in the brain. These findings suggest that MNCM may be useful as an AD preventive agent by targeting Aβ formation.

## 1. Introduction

Alzheimer’s disease (AD) is the most common form of dementia and a public health concern worldwide. AD is the fifth leading cause of death among people over 65 years old [1] and growth of AD prevalence is expected. In the USA, the care cost for dementia patients has been estimated at $290 billion, rendering a huge economic problem for society [2]. AD is a chronic neurodegenerative disorder that is characterized by the loss of cholinergic neurons, low levels of acetylcholine, and aggregation of neurotoxic amyloid beta plaque. Loss of cholinergic neurons located in the basal forebrain leads to reduced production of the neurotransmitter, acetylcholine, which is involved in memory and cognitive functions. Hence, inhibition of acetylcholine-degrading enzymes, cholinesterases (acetylcholinesterase (AChE) and butyrylcholinesterase (BChE)), may improve attention span and cognitive ability [3]. The accumulation of amyloid beta or beta amyloid (Aβ) peptides results from the cleavage of transmembrane amyloid precursor protein (APP) by β-site amyloid precursor protein cleaving enzyme 1 or beta-secretase 1 (BACE-1) as one of the AD hallmarks [4]. The Aβ peptides, either as Aβ_1–40_ or Aβ_1–42_, are secreted and aggregated as a dense senile plaque due to their hydrophobic properties. However, familial AD patients usually exhibit a higher ratio of Aβ_1–42_ in the brain, indicating that Aβ_1–42_ may be used as a marker for AD pathogenesis [5]. Therefore, besides cholinesterase inhibitors, BACE-1 inhibitors could be a target of interest for AD prevention.

Several epidemiological reports have documented that the consumption of fruits and vegetables may prevent or delay the onset of degenerative diseases, including AD and dementia [6,7]. Anthocyanins are flavonoids and occur in fruits and vegetables, mostly in berries. It has been shown that anthocyanins prevent streptozotocin-induced sporadic dementia of Alzheimer’s type by decreasing AChE activity in both the cerebral cortex and hippocampus of rats [8]. Furthermore, anthocyanins, including cyanidin-3-glucoside, delphinidin-3-glucoside, and petunidin-3-glucoside, suppressed BACE-1 expression in the hippocampal neurons of Aβ_1–42_-treated rats [9]. These data support the postulation that anthocyanin-rich foodstuffs may exert anti-AD properties and promote AD prevention and treatment.

The mulberry tree belongs to the family Moraceae. Three important mulberries are widely grown as *Morus alba*, *Morus rubra*, and *Morus nigra* [10]. Interestingly, *M. nigra* has been reported to have the highest amounts of anthocyanins compared to other species [10,11]. *M. nigra* is generally known as black mulberry. This plant is cultivated in Africa, South America, and Asian countries, including Thailand. Almost all parts of *M. nigra* are utilized for both food and pharmacological properties. Its leaves have been demonstrated to have antinociceptive, anti-inflammatory, and antidiabetic properties [12], while the fruits have historically been used as food because they are rich in nutrition elements, flavonols, and anthocyanins [11,13]. The fruits are also used in traditional medicine as they exert a wide range of health benefits, such as antimicrobial, anti-inflammatory, and antioxidative stress properties [12,14]. Evidence showed that compounds isolated from *M. alba* as artoindonesianin O and inethermulberrofuran C exhibited anti-AD properties [15,16]; however, little is known about the anthocyanin-rich *M. nigra*. Therefore, here, the anti-AD properties of *M. nigra* were investigated. A well-characterized *Morus* cf. *nigra* ‘Chiang Mai’ (MNCM) that is widely planted in Thailand was used in this study. The mulberry fruits of the mentioned cultivar were determined for their extraction conditions, phytochemical contents, antioxidative stress, and inhibitory activity against AChE, BChE, and BACE-1 in vitro. The extract was also determined for its anti-AD properties, targeting Aβ peptides in the adrenal phaeochromocytoma (PC12) neuronal cells and in a *Drosophila* model of AD. These flies were developed for studying potential therapeutic approaches since human APP and BACE-1 are co-expressed specifically in the central nervous system (CNS), representing the production of Aβ peptides in humans.

## 2. Results

### 2.1. Extraction Optimization of Morus cf. nigra ‘Chiang Mai’ (MNCM)

To optimize the extraction conditions regarding anti-AD properties of MNCM, aqueous ethanol was utilized as a solvent for anthocyanins extraction. First, the sample (30 mg/mL) was extracted with 0–100% (*v*/*v*) aqueous ethanol at 30°C for 30 min. The results suggested that acetylcholinesterase (AChE) inhibition was optimally achieved when extraction was performed under 0% (*v*/*v*) ethanol (or ultrapure water) (Table 1). Inhibition decreased with an increasing percentage of ethanol to 20% (*v*/*v*), and then completely diminished.

The effects of shaking time on AChE inhibition were then investigated by utilizing water extraction of MNCM. The shaking time varied from 0.5 to 6 h and was applied with fixed conditions of a 30 °C extraction temperature and 30 mg/mL extraction concentration. The results suggested that AChE inhibition was continuously elevated with an increased shaking time and achieved the significantly highest inhibition at the 2-h shaking time (Table 2). However, AChE inhibition started to decline after reaching this optimal shaking time.

The last parameter for MNCM extraction was the extraction temperature. The effect of temperature (30–90 °C) on AChE inhibition using water extraction conditions of a 2-h shaking time and 30 mg/mL extraction concentration was investigated. The results indicated that AChE inhibition increased with increasing extraction temperature and reached optimal inhibition at 50 °C (Table 3). However, when raising the extraction temperature above 50 °C, AChE inhibition declined to the lowest inhibition at 90 °C.

Thus, the optimized extraction conditions of MNCM to achieve the highest AChE inhibition were aqueous-based extraction (ultrapure water) using a 50 °C extraction temperature and 2-h shaking time.

### 2.2. Antioxidant Activities of MNCM Extract

Antioxidant activities were determined using MNCM extracted under optimized extraction conditions as mentioned above. Antioxidant activities determined by the 2,2-diphenyl-1-picrylhydrazyl (DPPH) radical scavenging assay suggested that MNCM extract exhibited scavenging activity of 0.40 ± 0.03 µmol TE/100 g DW, while the chelating ability of ferrous ion was 21.33 ± 0.35 µmol TE/g DW as investigated by the ferric ion reducing antioxidant power (FRAP) assay. The antioxidant capacity measured by the oxygen radical absorbance capacity (ORAC) assay was determined at 132.21 ± 8.88 µmol TE/g DW.

### 2.3. Phytochemical Analysis

It was found that MNCM extracted under optimized extraction conditions exhibited total phenolic contents (TPCs) of 6.93 ± 0.58 mg GAE/g DW. The only anthocyanin detected in MNCM extracted under acidic methanol was cyanidin, with a content of 233.77 ± 24.02 µg/g DW, while anthocyanins were detected as cyanidin-3-O-rutinoside or keracyanin (610.99 ± 9.17 µg/g DW) and cyanidin-3-O-glucoside or kuromanin (730.97 ± 3.61 µg/g DW) utilizing high performance liquid chromatography (HPLC) analysis (Figure S1).

### 2.4. MNCM Extract Inhibits Cholinesterase and BACE-1 Activities in Vitro

The MNCM extracted under optimized extraction conditions inhibited the key enzymes involved in AD, including AChE, BChE, and BACE-1, at different percentages. The AChE inhibitory activity of MNCM extract with 55.36 ± 4.02% inhibition was lower than BChE inhibition with 81.43 ± 4.56% inhibition at the final extract concentration of 5 mg/mL. Under the same extract concentration, BACE-1 inhibition was reported at 66.34 ± 5.32%.

### 2.5. MNCM Extract Prevents Aβ Peptide-Induced Toxicity and Promotes Neurite Outgrowth

**Figure 1:**
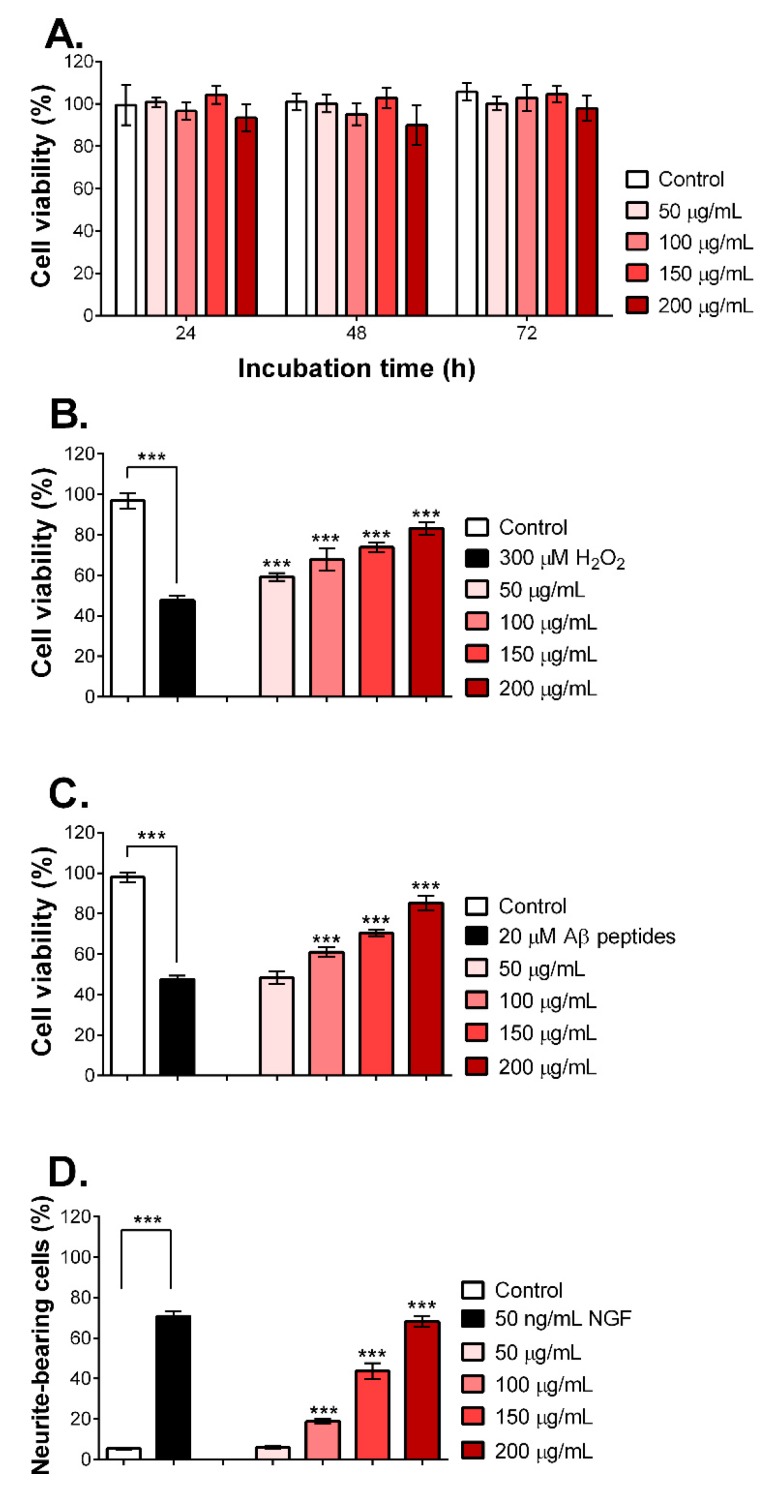
(**A**) Determination of safe doses of MNCM extract on the cell viability of PC12 cells after 24, 48, and 72 h of MNCM extract treatment, the percentage of cell viability is displayed. (**B**) Preventive effects of MNCM extract on H_2_O_2_-induced cell death, cells were pre-treated with the extract (50–200 µg/mL) for 24 h, then 300 µM of H_2_O_2_ was added for another 24 h. The percentage of cell viability is illustrated. (**C**) Preventive effects of MNCM aqueous extract on Aβ peptide-induced cell death, cells were pre-treated with MNCM extract (50–200 µg/mL) for 24 h, then 20 µM of Aβ peptides were added for another 24 h. The percentage of cell viability is illustrated. (**D**) Effects of MNCM extract (50–200 µg/mL) on neurite outgrowth compared with the nerve growth factor (NGF, 50 ng/mL). The data are represented as the percentage of neurite-bearing cells. The bar graphs are representative of three experiments and show mean ± standard deviation (SD). The one-way ANOVA followed by Tukey’s test was used to determine the differences between groups. ***, *p* < 0.001.

To investigate the neuroprotective effect of MNCM extract on PC12 neuronal cells, the cytotoxicity of MNCM extract was studied. PC12 cells were exposed with various concentrations of MNCM extract (50–200 µg/mL) for 24, 48, and 72 h. Results from the resazurin assay (Figure 1A) displayed that all concentrations of MNCM aqueous extract were not toxic to PC12 cells even after 72 h of treatment. We then selected these four concentrations for further analysis.

As mentioned above, MNCM is rich in anthocyanins and anthocyanidins, resulting in antioxidant activities. In addition, free radicals are also involved in the pathogenesis of AD [17]. Therefore, the protective effects of MNCM extract against H_2_O_2_, an oxidative stress inducer, were determined. Pre-treatment of PC12 cells with MNCM extracts (50–200 µg/mL) for 24 h significantly protected cells from oxidative stress-induced cell death in a dose-dependent manner compared with non-pretreated cells, as seen in Figure 1B, confirming the antioxidant activities in vitro.

It is well established that Aβ peptide-induced neuronal toxicity occurs via oxidative stress induction [18]. As illustrated in Figure 1C, PC12 cells were pre-treated with MNCM extract for 24 h before adding Aβ_25–35_ peptides. The Aβ_25–35_ peptides are widely used in AD study. Moreover, they have short fragments but retain active domains of Aβ_1–42_. In addition, the Aβ_25–35_ and Aβ_1–42_ peptides induce neural toxicity in a similar fashion [19]. Figure 1C shows that non-pretreated cells gave approximately 40% cell viability after exposure to Aβ_25–35_ peptides, whereas MNCM extract prevented Aβ peptide-induced toxicity in a dose-dependent manner, similar to Figure 1B. It seemed likely that the 200 µg/mL extract could diminish all adverse effects of Aβ peptides compared with the DI treatment.

Neurite outgrowth is a vital mechanism in neuronal growth and differentiation, and defects in the process might lead to neurodegenerative disorders like AD [20]. Therefore, we determined the effects of MNCM extract on neurite outgrowth. The results in Figure 1D and Figure S2 show that cells without nerve growth factor (NGF) or MNCM extract contained a lower percentage of neurite-bearing cells, whereas nerve growth factor (NGF) stimulated neurite outgrowth as previously reported. A dose-dependent manner of MNCM extract in stimulating neurite outgrowth was observed. Intriguingly, a high dose of MNCM extract at 200 µg/mL activated neurite outgrowth similar to the NGF-treated cells.

In conclusion, aqueous extract of MNCM was not toxic to PC12 cells, prevented H_2_O_2_ or Aβ peptide-induced cell death, and promoted neurite outgrowth.

### 2.6. MNCM Extract Reduces Aβ_1-42_ by Inhibiting BACE-1 Activity in a Drosophila Model of AD

**Figure 2:**
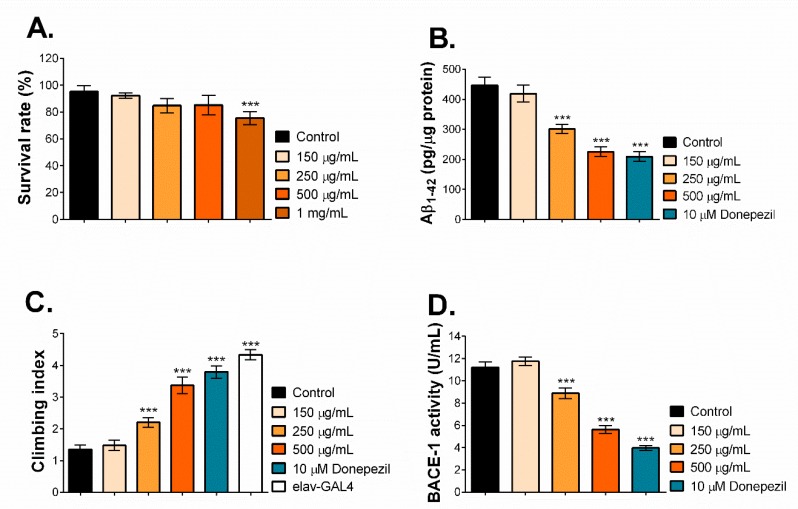
(**A**) Determination of safe doses of MNCM extract in fly larvae, the third-instar larvae were fed with MNCM extract (150 µg/mL–1 mg/mL). The surviving flies were counted within 5 days after the first eclosion and the percentage of the survival rate was calculated. (**B**) Effects of MNCM extract on the accumulation of Aβ_1–42_ peptides in fly brains. Flies were treated with MNCM extract (150–500 µg/mL) for 28 days, and after that fly heads were lysed and subjected for ELISA. (**C**) Effects of MNCM extract (150–500 µg/mL) on the locomotory coordination (climbing) of AD flies after 28 days of treatment. (**D**) Effects of MNCM extract on the BACE-1 activity in fly brains. Flies were treated with MNCM extract (150–500 µg/mL) for 28 days, then fly heads were lysed and subjected for BACE-1 activity determination. The data are representative of three replicates and show mean ± standard deviation (SD). The one-way ANOVA followed by Tukey’s test was used to determine the differences between groups. ***, *p* < 0.001.

To further investigate the anti-AD properties of MNCM extract in vivo, we employed a *Drosophila* model to our advantage by co-expressing human APP and BACE-1 specifically in the CNS of fly brains, thereby representing the amyloidogenic pathway. These short memory-deficient AD flies proved to be a useful tool to delineate the preventive effects of food or phytochemicals on the Aβ pathway [21]. First, we investigated safe doses of MNCM extract in *Drosophila* larvae. Larvae were exposed to MNCM extracts (0–1 mg/mL), and then the hatched flies were scored. As seen in Figure 2A, compared to the DI treatment, MNCM extracts up to 500 µg/mL were not toxic, whereas toxicity was observed at 1 mg/mL of MNCM extract. Thus, MNCM extracts at 150, 250, and 500 µg/mL were selected and used for further analysis.

It is known that the cleavage of APP by BACE-1 results in Aβ peptides as AD hallmarks. Hence, the flies were treated with MNCM extract at the indicated concentration from one day after eclosion, and donepezil, an AD drug, was used as the control. After 28 days, heads were collected, and the levels of Aβ_1–42_ peptides were quantified. The data showed that donepezil and 500 µg/mL of MNCM extract reduced Aβ_1-42_ peptide formation by approximately 2 fold compared with DI-treated flies (Figure 2B). A lesser reduction was also observed at 250 µg/mL of MNCM extract, while MNCM extract at 150 µg/mL was not potent enough to reduce Aβ_1–42_ formation, consistent with the cell study.

AD leads to a progressive decline in locomotory coordination. This ability can be measured by the climbing assay in *Drosophila*. Therefore, we tested whether MNCM extract ameliorated Aβ_1–42_-induced motor dysfunction in the AD flies. Using the same treatment as above, at day 28, flies were recorded for their climbing index. As shown in Figure 2C, the DI-treated flies representing AD exhibited an extremely reduced ability to climb compared to the AD-free flies (elav-GAL4), suggesting severe locomotory coordination possibly from high amounts of Aβ_1–42_ peptides (Figure 2B). Interestingly, the climbing index was rescued in a dose-dependent manner when flies were exposed to MNCM extracts at 250 and 500 µg/mL and donepezil.

To test whether MNCM extract acts as a BACE-1 inhibitor and leads to a reduction in Aβ_1–42_ peptides, fly brain lysates at day 28 of treatment were prepared and determined for BACE-1 activity. It was found that MNCM extract at 150 µg/mL and the DI control had the same BACE-1 activity (Figure 2D). However, flies treated with donepezil, and 250 and 500 µg/mL MNCM extract showed significantly decreased BACE-1 activity in AD fly brains. Donepezil is claimed to be a cholinesterase inhibitor, and its BACE-1 inhibitory activity has been documented [22].

In summary, aqueous extract of MNCM reduced Aβ_1–42_ formation and improved locomotor dysfunctions by inhibiting BACE-1 activity in the *Drosophila* model of AD.

## 3. Discussion

AD is a complex and progressive neurodegenerative disorder and an effective therapy is lacking. Therefore, the identification of novel AD therapeutic agents is urgently required. It is well-documented that oxidative stress and the expression of cholinesterases and BACE-1 play a vital role in AD initiation and progression [3,4,17]; thus, an ideal AD therapeutic agent should function against different AD pathogenic mechanisms [23]. Plants and their bioactive constituents are of great interest due to their safety and efficacy. Indeed, many plant-derived compounds, including phenolic acids and flavonoids, have been reported for their anti-AD properties toward oxidative stress, AChE, BChE, and BACE-1 activities [23,24].

Mulberry has been recognized to be rich in anthocyanins as members of the flavonoids, and especially for *M. nigra*. Therefore, this project aimed to study the anti-AD properties of aqueous extracts of *M.* cf. *nigra* ‘Chiang Mai’ fruit (MNCM), which is widely grown in Thailand. The major findings were (i) MNCM extract was rich in anthocyanins and anthocyanidins, especially cyanidin, kuromanin, and keracyanin, which are probably involved in antioxidative stress; (ii) MNCM extract exhibited up to 50% inhibitory activity against AChE, BChE, and BACE-1; (iii) MNCM extract protected neuronal cells from H_2_O_2_ or Aβ peptide-induced toxicity and promoted neurite outgrowth; and (iv) MNCM extract reduced Aβ_1-42_ peptides by inhibiting BACE-1 activity in a *Drosophila* model of AD.

Previous reports suggested that mulberry exhibited different degrees of phenolics and antioxidant activities depending on both internal factors (such as cultivars and stages of maturity) and external factors (such as detection methods and extraction conditions) [25,26]. Comparing water-extracted MNCM with the TPCs of 6.39 mg GAE/g DW, five cultivars of Korean mulberries (*M. alba*) extracted under 70% (*v*/*v*) aqueous ethanol exhibited lower TPCs, ranging from 2.2 to 2.6 mg GAE/g DW [25]. Besides, water-extracted MNCM also exhibited higher TPCs than methanolic-extracted *M. alba* collected in North Serbia, with the TPCs ranging from 1.05 to 2.16 mg GAE/g DW [27]. As for antioxidant activities, these values seemed to be greatly affected by both internal and external factors. In comparison to MNCM with ORAC activity of 132.21 ± 8.88 µmol TE/g DW, it was previously suggested that juices from different maturity stages of thornless blackberry exhibited ORAC activities ranging from 86.8–204.1 µmol TE/g DW, while those from red raspberry ranged of 40.8–114.9 µmol TE/g DW [28]. In the same study, juices from various cultivars of ripe strawberry exhibited ORAC activities of 120.8–172.3 µmol TE/g DW. The FRAP activity of MNCM (21.33 ± 0.35 µmol TE/g DW) was comparable to raspberry (28.11 µmol TE/g DW) and cranberry (22.41 µmol TE/g DW) extracted under a mixture of 70% (*v*/*v*) aqueous methanol (MeOH) and 70% (*v*/*v*) aqueous acetone (1:1, *v*/*v*) [29]. Interestingly, the FRAP activity of MNCM was higher than blackcurrant and blueberry (17.81 and 17.27 µmol TE/g DW, respectively) but lower than those of blackberry and black chokeberry (11.63 and 33.16 µmol TE/g DW, respectively) [29]. However, the DPPH radical scavenging activity (0.40 ± 0.03 µmol TE/100 g DW or approximately 0.44 µmol TE/100 g fresh weight with 90% moisture content) of MNCM was lower than blackberries, black mulberries (*M. nigra*), bilberries, and blackthorns ranging from 1.6–8.4 µmol TE/100 g frozen fruit extracted under acidified MeOH (0.1% HCl) [30].

Interestingly, keracyanin (60%) and kuromanin (38%) were the two main anthocyanins detected in mulberry [31,32]. Keracyanin (610.90 µg/g DW) and kuromanin (730.97 µg/g DW) detected in MNCM extract were in the range of those detected in Korean mulberries (30.6–486.7 µg keracyanin/g DW and 93.2–1364.9 µg kuromanin/g DW) [25]. The aqueous extract of MNCM provided good inhibitory activities against AChE, BChE, and BACE-1. It was previously reported that 18 commercially available mulberries (*M. alba*) in Poland, extracted under 80% (*v*/*v*) aqueous methanol, exhibited AChE inhibitory activity in the range of 2.6–37.9% [33]. However, this paper failed to indicate the extract concentration in the enzyme assay, and this cannot be used for comparison with MNCM extract. Considering the predominant anthocyanins and anthocyanidin detected in mulberry, cyanidin was able to inhibit AChE, with IC_50_ of 14.43 µM, while its BChE inhibitory activity was slightly higher [34]. Compared to cyanidin, its anthocyanin glycosides, including keracyanin and kuromanin, with insignificantly different cholinesterase inhibition, exhibited lower inhibitory activity against AChE and BChE [34]. However, an in vitro report on the BACE-1 inhibitory activity of these anthocyanidins and anthocyanidins remains unwritten.

After studying the anti-AD properties in vitro regarding AChE, BChE, and BACE-1, we also examined the anti-AD effects of MNCM extract on PC12 neuronal cells. As shown in Figure 1B,C, pre-treatment with MNCM extract evidently protected cells from H_2_O_2_ or Aβ peptide-induced toxicity. It may be possible that MNCM extract exerted its effective antioxidative properties based on the high amounts of anthocyanins and anthocyanidins as previously mentioned. Furthermore, it has been found that Aβ_25–35_ peptides cause PC12 apoptosis by triggering oxidative stress, lipid peroxidation, and intracellular calcium ([Ca^2+^]_i_), similar to that of H_2_O_2_ [35,36], indicating that Aβ_25–35_ peptides lead to mitochondrial dysfunction. Mitochondria is an organelle function in ATP synthesis and Ca^2+^ homeostasis, thus its impairment will provoke [Ca^2+^]_i_ release and the apoptotic protease-activating factor 1 (Apaf1)-mediated intrinsic apoptotic pathway. Indeed, Aβ_25–35_ peptides induced Apaf1-mediated cell death, while pre-treatment with ethanolic extract of Chinese *M. nigra* for 24 h followed by Aβ_25–35_ exposure downregulated Apaf1 [37]. Previous studies showed that cyanidin and kuromanin attenuate Aβ-induced PC12 neurotoxicity by maintaining mitochondrial stability [38]. Interestingly, MNCM extract was high in cyanidin, kuromanin, and keracyanin, indicating that MNCM extract may play a role in maintaining mitochondrial stability, which eventually leads to reduced Aβ_25–35_-mediated cell death. Additionally, kuromanin has been reported to reverse ethanol-induced inhibition of neurite outgrowth [39]. Neurite outgrowth is in neuron growth, and poor neurite outgrowth is observed in AD [20]. MNCM extract activated the neurite outgrowth of PC12 cells (Figure 1D). A high dose of MNCM extract at 200 µg/mL activated neurite outgrowth better than that of NGF-exposed cells. This could be because MNCM may enhance the expression of NGF as demonstrated in *M. fructus* [40].

To elucidate the anti-AD properties of MNCM extract in depth, a *Drosophila* co-expressing human APP and BACE-1 was employed. The fruit fly has emerged as a promising alternative model for AD drug screening since transgenic flies carrying AD-related genes demonstrated AD characteristics as in humans [41]. Flies also possess a unique approach for AD study because the elav-GAL4 driver can drive the expression of AD proteins in the brain at an early stage. This was in accordance with one of the present strategies for anti-AD agents to target at the earlier stages [42]. As illustrated in Figure 2B,C,D, MNCM extract inhibited BACE-1 activity, resulting in decreased Aβ_1–42_ peptides and improved locomotor functions in AD flies, in a dose-dependent fashion. The data raise the hypothesis that MNCM extract may penetrate the blood–brain barrier (BBB) and restrain BACE-1 function. It is important to consider that several neurotherapeutic agents worked well in the cell study but not in animal models since they failed to cross the BBB. BACE-1 is a rate-limiting enzyme responsible for amyloid peptide production, thereby making it ideal for AD therapy [43]. Anthocyanins and anthocyanidins are able to cross the BBB, especially kuromanin, and are located in the brain regions contributing to cognitive functions [44,45]. Thus, the present data support that MNCM could be further developed as a potential natural product for AD prevention by targeting BACE-1. Furthermore, in this study, MNCM was extracted by water, making it useful for further application as a functional food for neuroprotection.

## 4. Materials and Methods

### 4.1. Mulberry Collection and Preparation

*Morus* cf. *nigra* ‘Chiang Mai’ (MNCM) was sourced from the Queen Sirikit Department of Sericulture. The sample was identified based on the morphology and nuclear ribosomal internal transcribed spacer (nITS) (GenBank: MK946679.1) and deposited at the Bangkok Herbarium (BK), Bangkok, Thailand. The Herbarium voucher specimen is B. Jongruaysup et al. 12-1 (BK). Fruits of uniform color and ripening stage were selected and cleaned before being freeze-dried. Samples were then ground to a fine powder and extracted using ultrapure water (Smart2Pure 3 UV^TM^ Water Purification System, Thermo Fisher Scientific, Waltham, MA, USA) at 50 °C for 2 h.

### 4.2. In Vitro Antioxidant Activity

The in vitro antioxidant activity of the mulberry extract was performed from a well-established protocol for 2,2-diphenyl-1-picrylhydrazyl (DPPH) scavenging activity, oxygen radical absorbance capacity (ORAC), and ferric ion reducing antioxidant power (FRAP) assays [46,47,48,49].

### 4.3. Total Phenolic Contents, Anthocyanin, and Anthocyanidin Determination

Total phenolic contents (TPCs) were determined using Folin-Ciocalteu reagent as described by Thuphairo et al. 2019 [24]. Gallic acid was used as a reference matter, and the TPCs were reported as mg gallic acid equivalent (GAE)/g dried matter (DW) [24]. To determine anthocyanins and anthocyanidins, the mulberry powder was extracted under acidic conditions. The extracts were collected by filtering through a 0.45-μm polytetrafluoroethylene (PTFE) syringe filter. The HPLC analysis (an UtiMate HPLC with an HPG-3400SD pump and a photodiode array detector from DIONEX, Sunnyvale, CA, USA) was performed using Thermo Scientific Chromeleon Chromatography Data System (CDS) software (DIONEX, Sunnyvale, CA, USA) and a Reprosil-Pur ODS-3 column (250 mm × 4.6 mm, 5 µm from Dr. Maisch GmbH, Ammerbuch, Germany). For anthocyanidin analysis, a constant flow rate of 1 mL/min at ambient temperature was employed with an isocratic solvent of 82% Milli-Q water containing 0.4% (*v*/*v*) trifluoroacetic acid (TFA) (Solvent A) and 18% acetonitrile containing 0.4% (*v*/*v*) TFA (Solvent B) over 60 min. For anthocyanin analysis, a constant flow rate of 1 mL/min at ambient temperature was employed. The solvent system is shown in Table 4.

Samples were kept in the autosampler at 4 °C until injection (20 µL). The presence of anthocyanins and anthocyanidins was visualized at 525 and 530 nm, respectively. Anthocyanins (cyanidin-3-O-glucoside (kuromanin), cyanidin-3-O-rutinoside (keracyanin), cyanidin-3,5-O-diglucoside (cyanin), cyanidin-3-O-galactoside (idaein), pelargonidin-3,5-O-diglucoside (pelargonin), malvidin-3-O-galactoside (primulin), and petunidin-3-O-glucoside) and anthocyanidins (cyanidin, delphinidin, pelargonidin, peonidin, petunidin, and malvidin) were used as standards to identify anthocyanins and anthocyanidins in the sample by comparing their retention times (*R_t_*) and UV-vis spectral fingerprints. All chemicals were received from Sigma-Aldrich (St. Louis, MO, USA).

### 4.4. Determination of Cholinesterases and Beta-Secretase 1 (BACE-1) Inhibitory Activities

Inhibitory activity of MNCM extract against AChE was carried out as previously reported [24,50,51]. In brief, a mixture containing 20 ng of *Electrophorus electricus* AChE (1000 units/mg, 100 μL), 16 mM 5,5-dithio-bis-(2-nitrobenzoic acid) (DTNB, 10 μL), 0.8 mM acetylthiocholine (40 μL), and the extract (50 μL) were well-mixed. The initial velocity was measured at 412 nm using a microplate reader (Synergy^TM^ HT 96-well UV-visible spectrophotometer using Gen5 data analysis software from BioTek Instruments, Inc., Winooski, VT, USA). Percentage of inhibition was then calculated as follows:

(1) $$\%\;\mathrm{inhibition}=\left(1-\frac{B-b}{A-a}\right)\;\times \;100,$$

where *A* is the initial velocity of the reaction with enzyme, *a* is the initial velocity of the reaction without enzyme, *B* is the initial velocity of the enzyme reaction with extract, and *b* is the initial velocity of the reaction with extract but without enzyme.

Inhibitory activity of MNCM extract against BChE was determined similarly to AChE, except that 100 ng of equine serum BChE (≥10 units/mg protein, 100 μL) and 0.1 mM butyrylthiocholine (BTCh) were used as the reaction enzyme and substrate, respectively [24,50]. All chemicals and reagents for cholinesterase inhibitory activities were purchased from Sigma-Aldrich (St. Louis, MO, USA).

The BACE-1 activity was determined using a BACE-1 activity detection kit (Sigma-Aldrich, St. Louis, MO, USA) following the manufacturer’s instructions and expressed as the percentage of BACE-1 inhibition.

### 4.5. Cell Culture and Cytotoxicity Analysis by Resazurin Assay

The PC12 neuronal cells were obtained from the American Type Culture Collection (ATCC, Manassas, VA, USA) and cultured in Roswell Park Memorial Institute 1640 (RPMI1640), 10% (*v*/*v*) fetal bovine serum (FBS) (Thermo Fisher Scientific, Waltham, MA, USA) and 1% (*v*/*v*) penicillin-streptomycin (Sigma-Aldrich, St. Louis, MO, USA) at 37 °C in a humidified atmosphere containing 5% CO_2_. For cytotoxicity analysis, each well of a 96-well plate of 1 × 10^4^ exponentially growing PC12 cells was seeded and cultured overnight. Then, the cells were exposed to 50, 100, 150 and 200 µg/mL of MNCM extracts for 24, 48, and 72 h. At the indicated time, 20 µL of resazurin dye (CellTiter-Blue^®^, Promega, Madison, WI, USA) was added. One hour later, the fluorescence (emission = 585 nm, excitation = 570 nm) was measured. Cells treated with deionized water (DI) were used as a negative control.

### 4.6. Prevention of Hydrogen Peroxide (H_2_O_2_) and Aβ Peptide-Induced Toxicity

Each well of a poly-D-lysine (PDL)-coated 96-well plate of PC12 cells was plated and cultured overnight at a density of 2 × 10^4^ cells/mL. Then, the cells were treated with MNCM extracts for 24 h (50, 100, 150, and 200 µg/mL). After that, 300 µM of H_2_O_2_ (Merck, Darmstadt, Germany) or 20 µM of amyloid peptide (Aβ_25–35_) (Bachem, Bubendorf, Switzerland) were added. Forty-eight hours later, cell survival was measured by the resazurin assay, as mentioned above.

### 4.7. Determination of Neurite Outgrowth

Each well of a poly-D-lysine (PDL)-coated 6-well plate of PC12 cells was seeded and cultured overnight at a density of 1 × 10^4^ cells/mL. Cells were then treated with MNCM extracts at 50, 100, 150, and 200 µg/mL in RPMI1640 with 0.5% FBS for one week, and the medium was changed every two days. The nerve growth factor (NGF) (Sigma-Aldrich, St. Louis, MO, USA) at 50 ng/mL was used as a positive control. Before analysis under a phase-contrast microscope, the neurite-bearing cells were fixed with 4% paraformaldehyde. The percentage of neurite-bearing cells was expressed. At least three independent experiments were carried out.

### 4.8. Drosophila Stocks and Culture

Fly stocks (elav-GAL4 (8760) and UAS-APP-BACE-1) (33798)) were obtained from the Bloomington Stock Center at Indiana University. After eclosion, F1 progeny flies obtained from the mating between elav-GAL4 and UAS-APP-BACE-1 were cultured on Formula 4-24 blue^®^ medium (Carolina, Burlington, NC, USA) containing MNCM extract or DI (control) at 28 °C for 28 days. Flies were transferred to fresh media every 2–3 days. The *Drosophila* study was approved by Mahidol University-Institute Animal Care and Use Committee (MU-IACUC) (COA.No.MU-IACUC 2018/022).

### 4.9. Climbing Assay

The assay was determined following the published method [52]. At day 28 after treatment, 20 to 30 flies were placed in a transparent tube. After tapping, their rate of climb to the top of the tube was recorded and analyzed. At least three independent experiments were performed.

### 4.10. Quantification of Aβ Peptide by Enzyme-Linked Immunosorbent Assay (ELISA)

Quantification of Aβ peptide was performed as reported with slight modification [53]. The heads of F1 progeny flies at day 28 were collected and homogenized in 5 M guanidine-HCl containing 2X Halt protease inhibitor cocktail (Thermo Fisher Scientific, Waltham, MA, USA). Then, the protein concentration was measured using the BCA protein assay kit (Thermo Fisher Scientific, Waltham, MA, USA). Before sample loading, a serial dilution of supernatants was made with ELISA diluent buffer containing protease inhibitor cocktail. After following the manufacturer’s instructions (human Aβ_42_ ELISA kit (Thermo Fisher Scientific, Waltham, MA, USA)) the samples were measured at 450 nm. The concentration of Aβ_1-42_ peptides was calculated and compared with standard recombinant human Aβ_1-42_.

### 4.11. Determination of BACE-1 Activity in Fly Brain Lysate

The heads of F1 progeny flies at day 28 were collected and homogenized in T-PER tissue protein extraction reagent (Thermo Fisher Scientific, Waltham, MA, USA). Then, the protein concentration and BACE-1 activity were measured as mentioned above within the same day.

## Figures and Tables

**Table 1:** Effects of different percentages of aqueous ethanol on MNCM extraction regarding AChE inhibition.

Independent Variable (Solvents, % (*v*/*v*) Aqueous Ethanol)	Dependent Variable % AChE Inhibition	Controlled Variables
0% (*v*/*v*) (ultrapure water)	40.29 ± 1.27 ^a^	Extraction temperature 30 °C Shaking time 30 min Extraction concentration 30 mg/mL
20% (*v*/*v*)	27.07 ± 1.36 ^b^	
40% (*v*/*v*)	ND *	
60% (*v*/*v*)	ND *	
80% (*v*/*v*)	ND *	
100% (*v*/*v*) (absolute ethanol)	ND *	

Values expressed are mean ± standard deviation (SD) of triplicate experiments (n = 3). Lower letter case indicates significant differences in each column at *p* < 0.05 calculated by one-way analysis of variance (ANOVA) and Duncan’s multiple comparison test. * ND = not detected.

**Table 2:** Effects of different shaking times on MNCM extraction regarding AChE inhibition.

Independent Variable (Shaking Time, h)	Dependent Variable % AChE Inhibition	Controlled Variables
0.5	27.63 ± 0.42 ^c^	Extraction temperature 30 °C Extraction solvent of water Extraction concentration 30 mg/mL
1	33.63 ± 0.97 ^b^	
2	43.35 ± 3.32 ^a^	
4	30.18 ± 1.15 ^c^	
6	28.41 ± 0.58 ^c^	

Values expressed are mean ± standard deviation (SD) of triplicate experiments (n = 3). Lower letter case indicates significant differences in each column at *p* < 0.05 calculated by one-way analysis of variance (ANOVA) and Duncan’s multiple comparison test.

**Table 3:** Effect of different temperatures on MNCM extraction regarding AChE inhibition.

Independent Variable (Temperature, °C)	Dependent Variable % AChE Inhibition	Controlled Variables
30	27.89 ± 1.36 ^b^	Extraction solvent of water Shaking time of 2 h Extraction concentration 30 mg/mL
50	32.20 ± 2.67 ^a^	
70	23.66 ± 2.16 ^c^	
90	11.29 ± 0.18 ^d^	

Values expressed are mean ± standard deviation (SD) of triplicate experiments (n = 3). Lower letter case indicates significant differences in each column at *p* < 0.05 calculated by one-way analysis of variance (ANOVA) and Duncan’s multiple comparison test.

**Table 4:** Solvent system of anthocyanin analysis using HPLC.

Time (min)	Solvent A	Solvent B
0	88	12
6	88	12
8	85	15
25	85	15
25	88	12
30	88	12

Solvent A = Milli-Q water containing 0.4% (*v*/*v*) TFA; solvent B = acetonitrile containing 0.4% (*v*/*v*) TFA.

## Supplementary Materials

The following are available online at https://www.mdpi.com/1420-3049/25/8/1837/s1, Figure S1: High-performance liquid chromatography (HPLC) chromatograms of (A.) cyanidin chloride standard (B.) kuromanin and keracyanin standard (C.) anthocyanidin analysis of MNCM extract and (D.) anthocyanin analysis of MNCM extract. The retention times (*R_t_*) of cyanidin chloride, kuromanin and keracyanin in MNCM extract was also indicated; Figure S2: Representative images from the neurite outgrowth study showing neurite-bearing cells of (A.) control, (B.) MNCM extract-treated cells at 50 µg/mL, (C.) MNCM extract-treated cells at 100 µg/mL, (D.) MNCM extract-treated cells at 150 µg/mL, (E.) MNCM extract-treated cells at 200 µg/mL, and (F.) NGF-treated cells at 50 ng/mL.

## References

[1] Wang H., Naghavi M., Allen C., Barber R.M., A Bhutta Z., Carter A., Casey D.C., Charlson F., Chen A.Z., Coates M.M. (2016). Global, regional, and national life expectancy, all-cause mortality, and cause-specific mortality for 249 causes of death, 1980–2015: A systematic analysis for the Global Burden of Disease Study 2015. Lancet.

[2] Alzheimer’s association (2015). Alzheimer’s disease facts and figures. Alzheimer’s Dement.

[3] Ferreira-Vieira T.H., Guimaraes I.M., Silva F.R., Ribeiro F. (2016). Alzheimer’s Disease: Targeting the Cholinergic System. Curr. Neuropharmacol..

[4] Hardy J., Selkoe D.J. (2002). The amyloid hypothesis of Alzheimer’s disease: Progress and problems on the road to therapeutics. Science.

[5] Citron M., Diehl T.S., Gordon G., Biere A.L., Seubert P., Selkoe D.J. (1996). Evidence that the 42- and 40-amino acid forms of amyloid β protein are generated from the β-amyloid precursor protein by different protease activities. Proc. Natl. Acad. Sci. USA.

[6] Dai Q., Borenstein A.R., Wu Y., Jackson J.C., Larson E.B. (2006). Fruit and Vegetable Juices and Alzheimer’s Disease: The Kame Project. Am. J. Med..

[7] Loef M., Walach H. (2012). Fruit, vegetables and prevention of cognitive decline or dementia: A systematic review of cohort studies. J. Nutr. Health Aging.

[8] Pacheco S.M., Soares M.S.P., Gutierres J.M., Gerzson M.F.B., Carvalho F.B., Azambuja J.H., Schetinger M.R.C., Stefanello F.M., Spanevello R.M. (2018). Anthocyanins as a potential pharmacological agent to manage memory deficit, oxidative stress and alterations in ion pump activity induced by experimental sporadic dementia of Alzheimer’s type. J. Nutr. Biochem..

[9] Badshah H., Kim T.H., Kim M.O. (2015). Protective effects of Anthocyanins against Amyloid beta-induced neurotoxicity in vivo and in vitro. Neurochem. Int..

[10] Özgen M., Serce S., Kaya C. (2009). Phytochemical and antioxidant properties of anthocyanin-rich *Morus nigra* and *Morus rubra* fruits. Sci. Hortic..

[11] Chen H., Yu W., Chen G., Meng S., Xiang Z., He N. (2017). Antinociceptive and Antibacterial Properties of Anthocyanins and Flavonols from Fruits of Black and Non-Black Mulberries. Molecules.

[12] Lim S.H., Choi C.-I. (2019). Pharmacological Properties of *Morus nigra L*. (Black Mulberry) as A Promising Nutraceutical Resource. Nutrients.

[13] Koyuncu F., Cetinbas M., Ibrahim E. (2014). Nutritional constituents of wild–grown black mulberry (*Morus nigra L*.). J. Appl. Bot. Food Qual..

[14] Imran M., Khan H., Shah M., Khan R., Khan F. (2010). Chemical composition and antioxidant activity of certain *Morus* species. J. Zhejiang Univ. Sci. B.

[15] Qiao A., Wang Y., Zhang W., He X. (2015). Neuroprotection of Brain-Targeted Bioactive Dietary Artoindonesianin O (AIO) from Mulberry on Rat Neurons as a Novel Intervention for Alzheimer’s Disease. J. Agric. Food Chem..

[16] Xia C.-L., Tang G., Guo Y.-Q., Xu Y.-K., Huang Z.-S., Yin S. (2019). Mulberry Diels-Alder-type adducts from *Morus alba* as multi-targeted agents for Alzheimer’s disease. Phytochemistry.

[17] Chen Z., Zhong C. (2014). Oxidative stress in Alzheimer’s disease. Neurosci. Bull..

[18] Butterfield D.A., Boyd-Kimball D. (2018). Oxidative Stress, Amyloid-β Peptide, and Altered Key Molecular Pathways in the Pathogenesis and Progression of Alzheimer’s Disease. J. Alzheimer’s Dis..

[19] Frozza R.L., Horn A.P., Hoppe J.B., Simao F., Gerhardt D., Comiran R.A., Salbego C.G. (2009). A comparative study of beta-amyloid peptides Abeta1–42 and Abeta25–35 toxicity in organotypic hippocampal slice cultures. Neurochem. Res..

[20] Dowjat W.K., Wisniewski T., Efthimiopoulos S., Wisniewski H.M. (1999). Inhibition of neurite outgrowth by familial Alzheimer’s disease-linked presenilin-1 mutations. Neurosci. Lett..

[21] Wang X., Kim J.-R., Lee S.-B., Kim Y.-J., Jung M.Y., Kwon H.W., Ahn Y.-J. (2014). Effects of curcuminoids identified in rhizomes of *Curcuma longa* on BACE-1 inhibitory and behavioral activity and lifespan of Alzheimer’s disease Drosophila models. BMC Complement. Altern. Med..

[22] Liu R., Liu Y.C., Meng J., Zhu H., Zhang X. (2017). A microfluidics-based mobility shift assay to identify new inhibitors of beta-secretase for Alzheimer’s disease. Anal. Bioanal. Chem..

[23] Mancini F., De Simone A., Andrisano V. (2011). Beta-secretase as a target for Alzheimer’s disease drug discovery: An overview of in vitro methods for characterization of inhibitors. Anal. Bioanal. Chem..

[24] Thuphairo K., Sornchan P., Suttisansanee U. (2019). Bioactive Compounds, Antioxidant Activity and Inhibition of Key Enzymes Relevant to Alzheimer’s Disease from Sweet Pepper (*Capsicum annuum*) Extracts. Prev. Nutr. Food Sci..

[25] Bae S.-H., Suh H. (2007). Antioxidant activities of five different mulberry cultivars in Korea. LWT.

[26] Oki T., Kobayashi M., Nakamura T., Okuyama A., Masuda M., Shiratsuchi H., Suda I. (2006). Changes in Radical-scavenging Activity and Components of Mulberry Fruit During Maturation. J. Food Sci..

[27] Natić M., Dabić D.Č, Papetti A., Akšić M.F., Ognjanov V., Ljubojević M., Tešić Ž. (2015). Analysis and characterisation of phytochemicals in mulberry (*Morus alba L*.) fruits grown in Vojvodina, North Serbia. Food Chem..

[28] Wang S.Y., Lin H.-S. (2000). Antioxidant activity in fruits and leaves of blackberry, raspberry, and strawberry varies with cultivar and developmental stage. J. Agric. Food Chem..

[29] Kim J.-S. (2018). Antioxidant Activities of Selected Berries and Their Free, Esterified, and Insoluble-Bound Phenolic Acid Contents. Prev. Nutr. Food Sci..

[30] Ştefănuţ M.N., Căta A., Pop R., Mosoarca C., Zamfir A.D. (2011). Anthocyanins HPLC-DAD and MS Characterization, Total Phenolics, and Antioxidant Activity of Some Berries Extracts. Anal. Lett..

[31] Pawlowska A.M., Oleszek W., Braca A. (2008). Quali-quantitative Analyses of Flavonoids of *Morus nigra L*. and *Morus alba L*. (Moraceae) Fruits. J. Agric. Food Chem..

[32] Qin C., Li Y., Niu W., Ding Y., Zhang R., Shang X. (2010). Analysis and characterisation of anthocyanins in mulberry fruit. Czech. J. Food Sci..

[33] Polumackanycz M., Sledzinski T., Goyke E., Wesolowski M., Viapiana A. (2019). A Comparative Study on the Phenolic Composition and Biological Activities of *Morus alba L*. Commercial Samples. Molecules.

[34] Szwajgier D. (2014). Anticholinesterase Activities of Selected Polyphenols—A Short Report. Pol. J. Food Nutr. Sci..

[35] Ye J., Meng X., Yan C., Wang C. (2009). Effect of Purple Sweet Potato Anthocyanins on β-Amyloid-Mediated PC-12 Cells Death by Inhibition of Oxidative Stress. Neurochem. Res..

[36] Hong H., Liu G.-Q. (2004). Protection against hydrogen peroxide-induced cytotoxicity in PC12 cells by scutellarin. Life Sci..

[37] Song N., Yang H., Pang W., Qie Z., Lu H., Tan L., Li H., Sun S., Lian F., Qin C. (2014). Mulberry extracts alleviate abeta 25-35-induced injury and change the gene expression profile in PC12 cells. Evid Based Complement. Alternat Med..

[38] Zheng Z.-C., Cho N.C., Wang Y., Fu X.-T., Li D.-W., Wang K., Wang X.-Z., Li Y., Sun B.-L., Yang X.-Y. (2016). Cyanidin suppresses amyloid beta-induced neurotoxicity by inhibiting reactive oxygen species-mediated DNA damage and apoptosis in PC12 cells. Neural Regen. Res..

[39] Chen G., Bower K.A., Xu M., Ding M., Shi X., Ke Z., Luo J. (2009). Cyanidin-3-Glucoside Reverses Ethanol-Induced Inhibition of Neurite Outgrowth: Role of Glycogen Synthase Kinase 3 Beta. Neurotox. Res..

[40] Kim H.G., Oh M.S. (2012). Memory-enhancing effect of Mori Fructus via induction of nerve growth factor. Br. J. Nutr..

[41] Pandey U.B., Nichols C.D. (2011). Human disease models in Drosophila melanogaster and the role of the fly in therapeutic drug discovery. Pharmacol. Rev..

[42] Chintamaneni M., Bhaskar M. (2012). Biomarkers in Alzheimer’s disease: A review. ISRN Pharmacol..

[43] Ghosh A.K., Gemma S., Tang J. (2008). beta-Secretase as a therapeutic target for Alzheimer’s disease. Neurotherapeutics.

[44] Fornasaro S., Ziberna L., Gasperotti M., Tramer F., Vrhovšek U., Mattivi F., Passamonti S. (2016). Determination of cyanidin 3-glucoside in rat brain, liver and kidneys by UPLC/MS-MS and its application to a short-term pharmacokinetic study. Sci. Rep..

[45] Afzal M., Redha A., AlHasan R. (2019). Anthocyanins Potentially Contribute to Defense against Alzheimer’s Disease. Molecules.

[46] Benzie I., Strain J. (1996). The Ferric Reducing Ability of Plasma (FRAP) as a Measure of “Antioxidant Power”: The FRAP Assay. Anal. Biochem..

[47] Fukumoto L.R., Mazza G. (2000). Assessing Antioxidant and Prooxidant Activities of Phenolic Compounds. J. Agric. Food Chem..

[48] Ou B., Hampsch-Woodill M., Prior R.L. (2001). Development and validation of an improved oxygen radical absorbance capacity assay using fluorescein as the fluorescent probe. J. Agric. Food Chem..

[49] Sripum C., Kukreja R.K., Charoenkiatkul S., Kriengsinyos W., Suttisansanee U. (2017). The effect of extraction conditions on antioxidant activities and total phenolic contents of different processed Thai Jasmine rice. Int. Food Res. J..

[50] Jung H.A., Min B.S., Yokozawa T., Lee J.-H., Kim Y.S., Choi J.S. (2009). Anti-Alzheimer and antioxidant activities of *Coptidis Rhizoma* alkaloids. Biol. Pharm. Bull..

[51] Nantakornsuttanan N., Thuphairo K., Kukreja R.K., Charoenkiatkul S., Suttisansanee U. (2016). Anti-cholinesterase inhibitory activities of different varieties of chili peppers extracts. Int. Food Res. J..

[52] Jantrapirom S., Piccolo L.L., Yoshida H., Yamaguchi M. (2018). A new Drosophila model of Ubiquilin knockdown shows the effect of impaired proteostasis on locomotive and learning abilities. Exp. Cell Res..

[53] Sofola-Adesakin O., Khericha M., Snoeren I., Tsuda L., Partridge L. (2016). pGluAbeta increases accumulation of Abeta in vivo and exacerbates its toxicity. Acta Neuropathol. Commun..

