# Supplementary material for: Mulberry Fruit Cultivar ‘Chiang Mai’ Prevents Beta-Amyloid Toxicity in PC12 Neuronal Cells and in a Drosophila Model of Alzheimer’s Disease
Source: Molecules. 2020 Apr 15;25(8):1837. doi: 10.3390/molecules25081837 (PMC7221829; doi:10.3390/molecules25081837)
Supplement: Supplementary file 1 [file molecules-25-01837-s001.pdf]

Supplementary Figure 1:

High-performance liquid chromatography (HPLC) chromatograms of (A.) cyanidin chloride standard (B.) kuromanin and keracyanin standard (C.) anthocyanidin analysis of MNCM extract and (D.) anthocyanin analysis of MNCM extract. The retention times (R_t) of cyanidin chloride, kuromanin and keracyanin in MNCM extract was also indicated

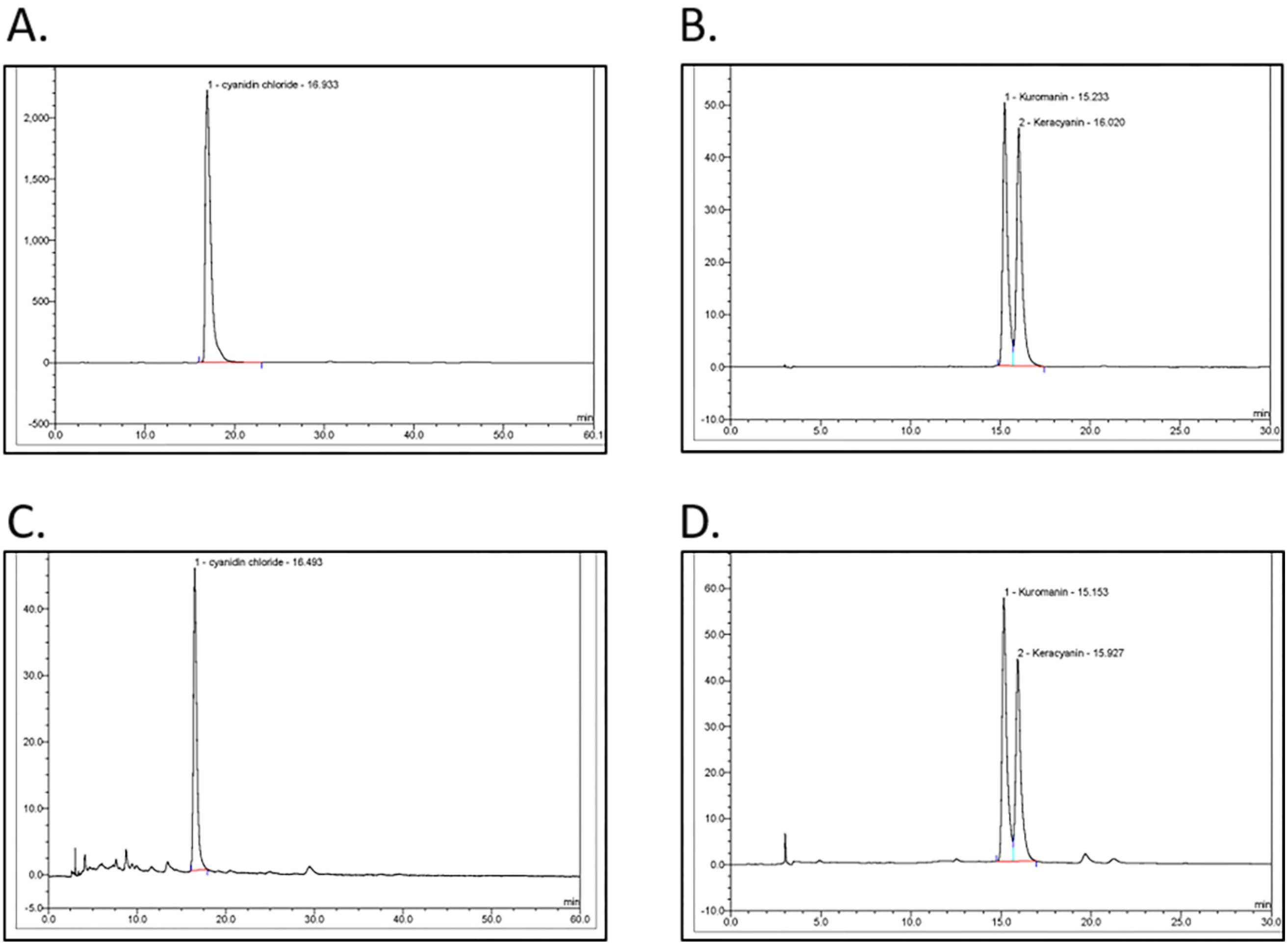

Supplementary Figure 2:

Representative images from the neurite outgrowth study showing neurite-bearing cells of (A.) control, (B.) MNCM extract-treated cells at 50 μg/mL, (C.) MNCM extract-treated cells at 100 μg/mL, (D.) MNCM extract-treated cells at 150 μg/mL, (E.) MNCM extract-treated cells at 200 μg/mL, and (F.) NGF-treated cells at 50 ng/mL

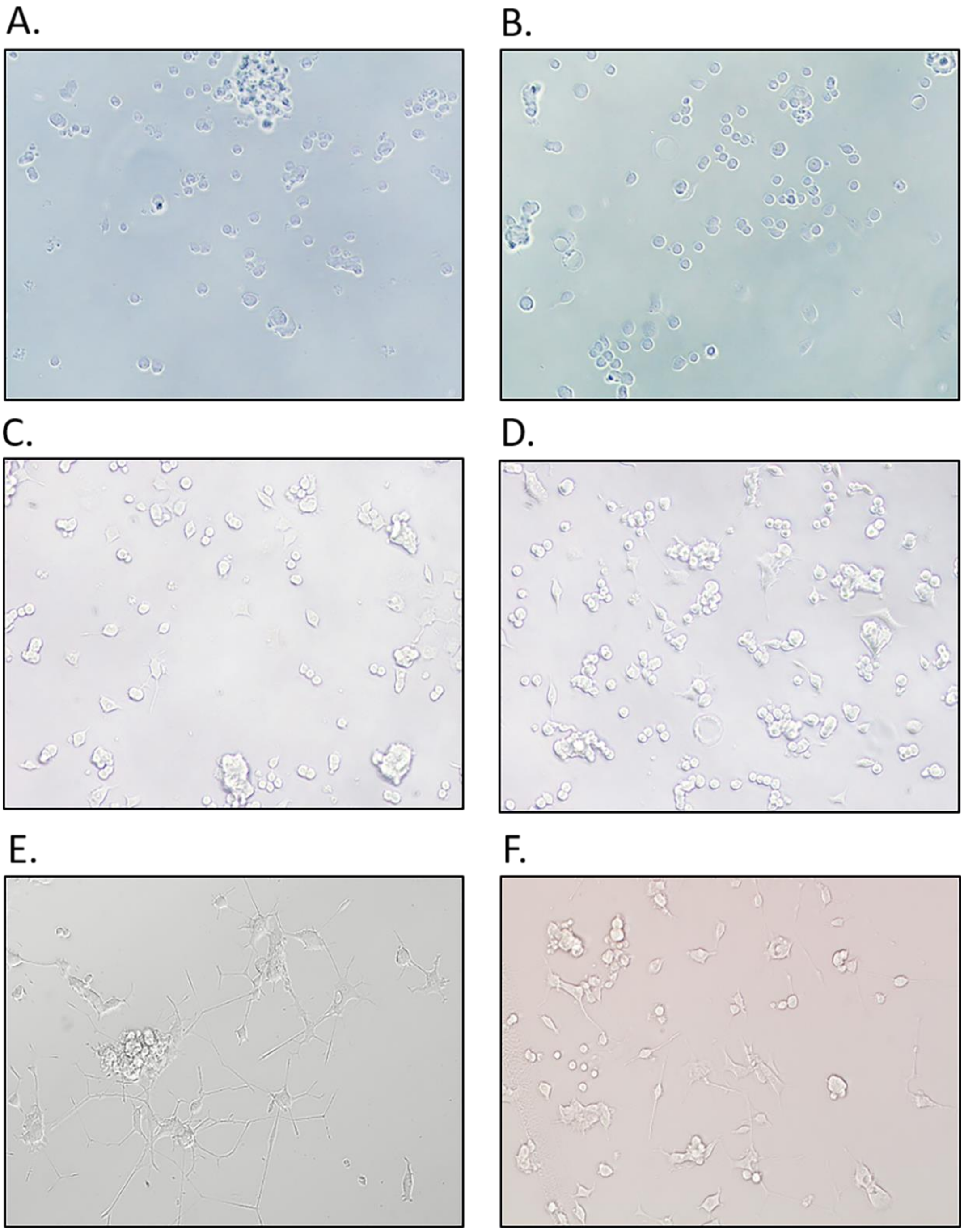
A.
B.
C.
D.
E.
F.
